# Crystal structure and Hirshfeld surface analysis of (*E*)-2-cyano-*N*′-(3,4,5-tri­meth­oxy­benzyl­idene)acetohydrazide

**DOI:** 10.1107/S2056989025003913

**Published:** 2025-05-09

**Authors:** Subramani Uma Maheswari, Srinivasan Senthilkumar, Sivashanmugam Selvanayagam

**Affiliations:** a Department of Chemistry Annamalai University, Annamalainagar Chidambaram 608 002 India; b Department of Science and Humanities, Rathinam Technical Campus, Coimbatore 641 021, India; c PG & Research Department of Physics, Government Arts College, Melur 625 106, India; University of Neuchâtel, Switzerland

**Keywords:** benzohydrazine, inter­molecular hydrogen bonds, Hirshfeld surface analysis, crystal structure

## Abstract

In the title compound, C_13_H_15_N_3_O_4_, the 2-cyano-*N*′-methyl­ideneacetohydrazide moiety and the trimeth­oxy phenol ring form a dihedral angle of 13.8 (1)°. Inter­molecular N—H⋯O and C—H⋯O hydrogen bonds are observed. The inter­molecular inter­actions were qu­anti­fied and analysed using Hirshfeld surface analysis, revealing that H⋯H inter­actions contribute most to the crystal packing (38.3%).

## Abstract 2

In the title compound, the crystal packing features strong N—H⋯O hydrogen bonds, which form *C*(4) chain-motifs.

## 1. Chemical context

Hydrazones have been found to show various biological properties, including anti­convulsant (Angelova *et al.*, 2016 ▸), anti­fungal (Ozdemir *et al.*, 2008 ▸) and anti­tumoral (Parlar *et al.*, 2018 ▸). In the present work, the synthesis, structural and computational studies of (*E*)-2-cyano-*N*′-(3,4,5-tri­meth­oxy­benzyl­idene)acetohydrazide, (I), are reported.
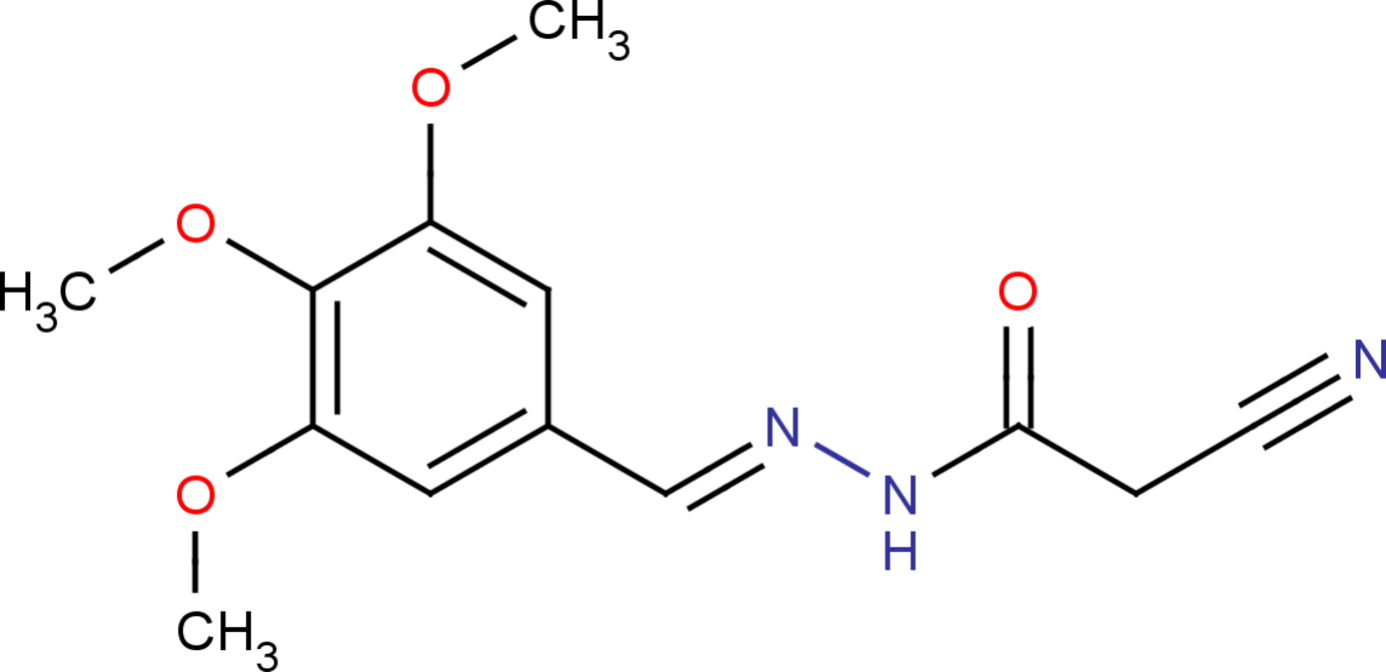


## 2. Structural commentary

**Figure 1:**
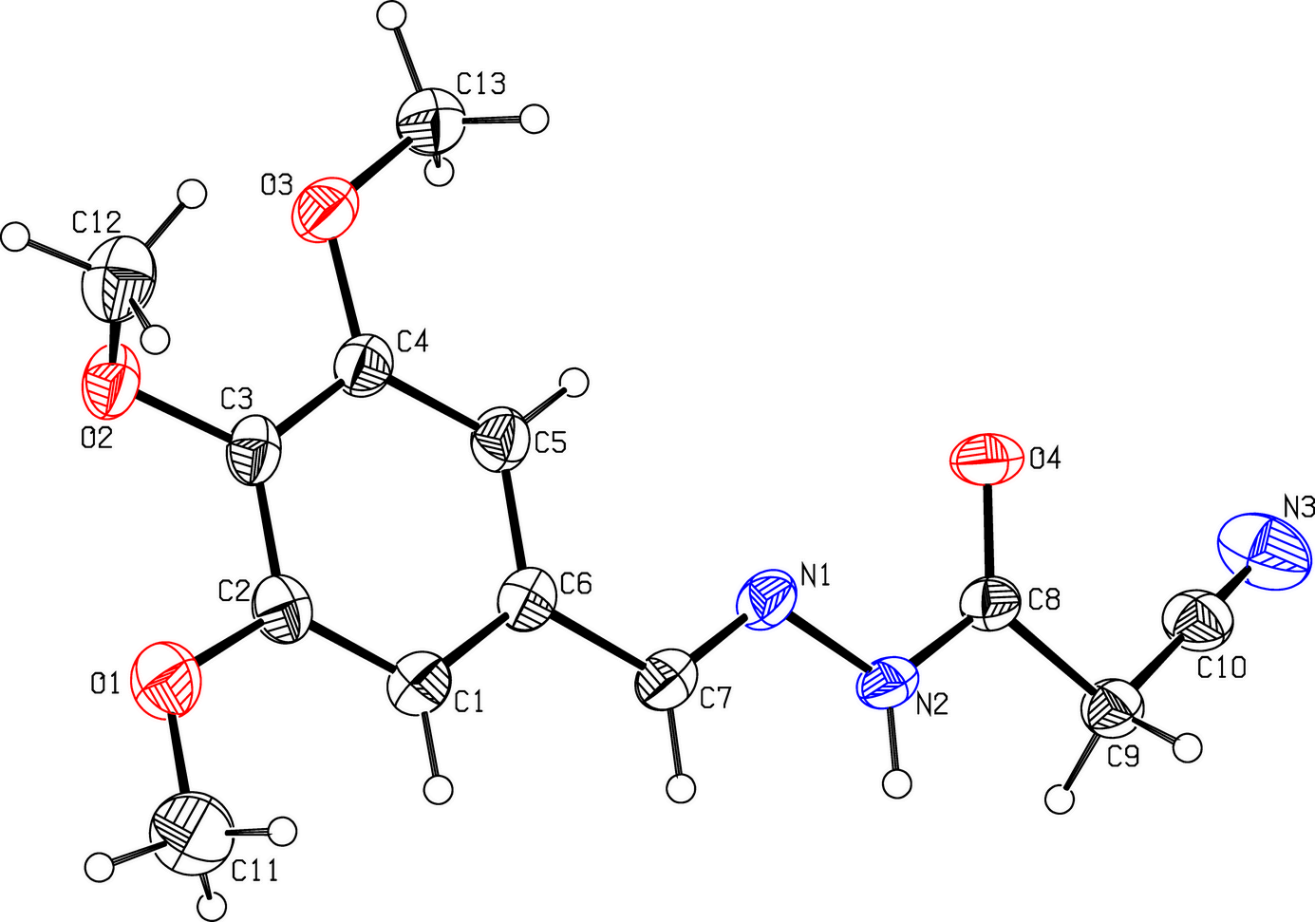
A view of the mol­ecular structure of compound (I), showing the atom labelling. Displacement ellipsoids are drawn at the 30% probability level.

The mol­ecular structure of (I) is displayed in Fig. 1 ▸. The phenyl ring (C1–C6) is planar with a maximum deviation of 0.008 (2) Å for atom C6 and its attached meth­oxy atoms O1, C11, O2, C12, O3 and C13 deviate by 0.023 (2), −0.169 (4), 0.133 (2), −1.089 (3), 0.010 (2) and 0.056 (3) Å, respectively. The 2-cyano-*N*′-methyl­ideneacetohydrazide moiety (C7/N1/N2/C8/O4/C9/C10/N3) is nearly planar with a maximum deviation of 0.280 (3) Å for atom N3. This moiety forms a dihedral angle of 13.8 (1)° with the trimeth­oxy phenyl ring.

## 3. Supra­molecular features

**Figure 2:**
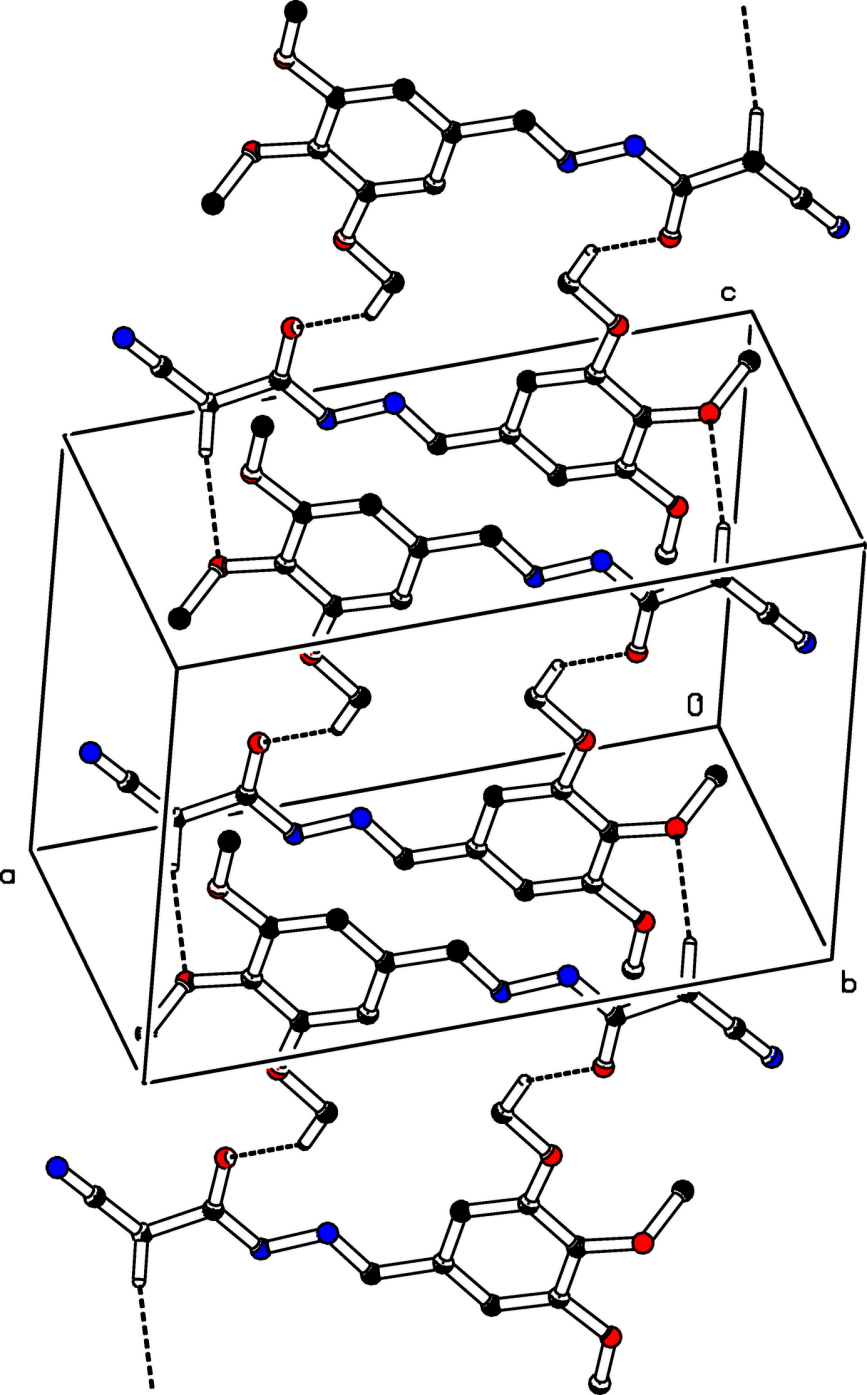
A view of the dimeric arrangement through O—H⋯O hydrogen bonds

**Figure 3:**
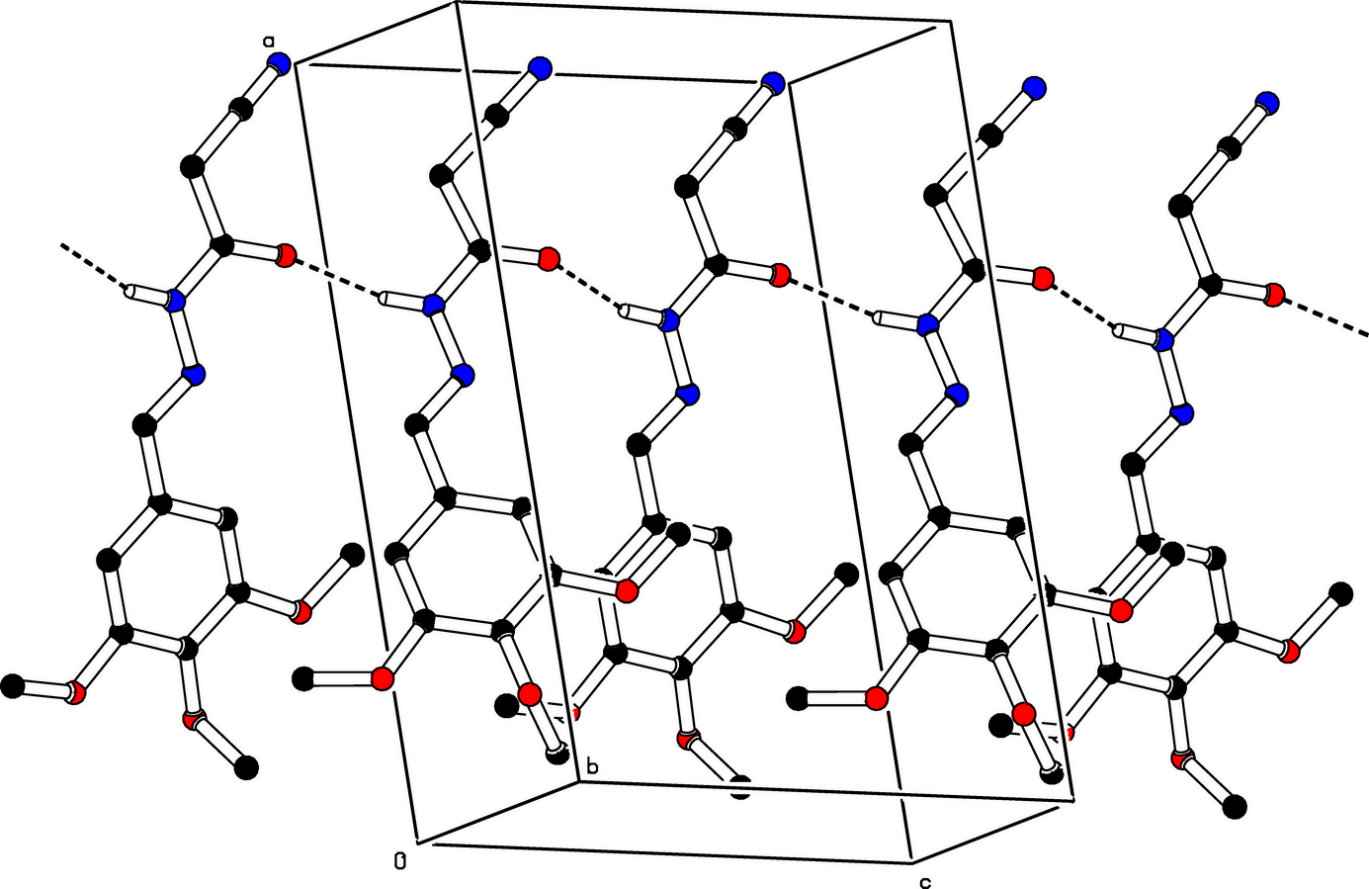
The crystal packing of the title compound (I) viewed along the *b* axis. The N—H⋯O and O—H⋯O inter­molecular hydrogen bonds are shown as dashed lines. For clarity, H atoms not involved in hydrogen bonds have been omitted.

In the crystal, mol­ecules associate pairwise by C9—H9*B*⋯O2^i^ and C13—H13*A*⋯O4^i^ hydrogen bonds (Table 1 ▸) into inversion dimers with an 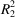 (22) graph-set motifs (Etter *et al.*, 1990 ▸), as shown in Fig. 2 ▸. The mol­ecules are further linked into a *C*(4) chain motif by N2—H2⋯O4^iii^ hydrogen bonds running parallel to [0
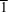
0] (Table 1 ▸; Fig. 3 ▸).

## 4. Hirshfeld surface analysis

**Figure 4:**
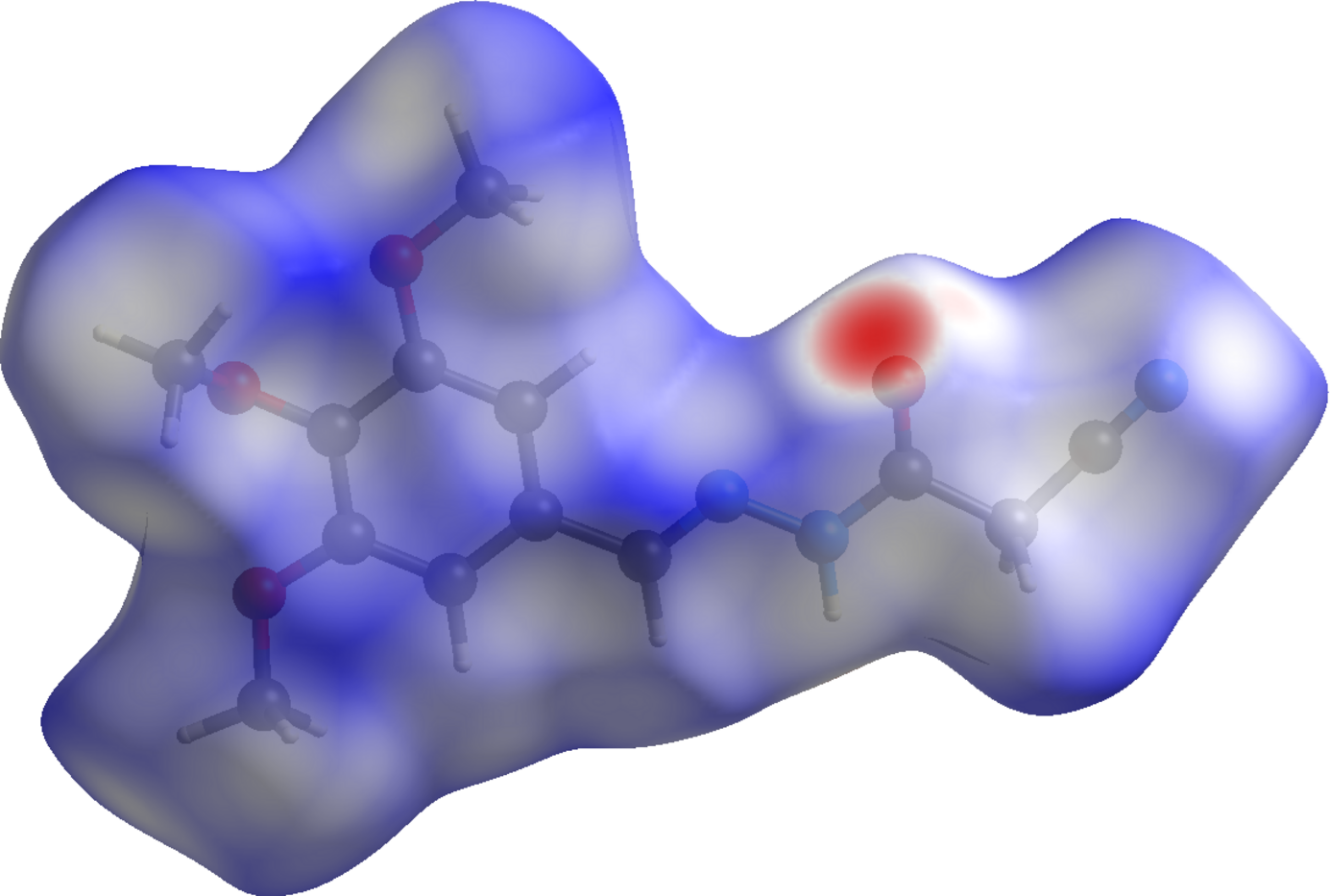
A view of the Hirshfeld surface mapped over *d*_norm_.

To further characterize the inter­molecular inter­actions, we carried out a Hirshfeld surface (HS) analysis (Spackman & Jayatilaka, 2009 ▸) using *Crystal Explorer 21* (Spackman *et al.*, 2021 ▸) The HS mapped over *d*_norm_ is illustrated in Fig. 4 ▸ where the deep-red spot occurs at O4 and this oxygen atom is responsible for intermolecular N—H⋯O and C—H⋯O hydrogen bonds.

**Figure 5:**
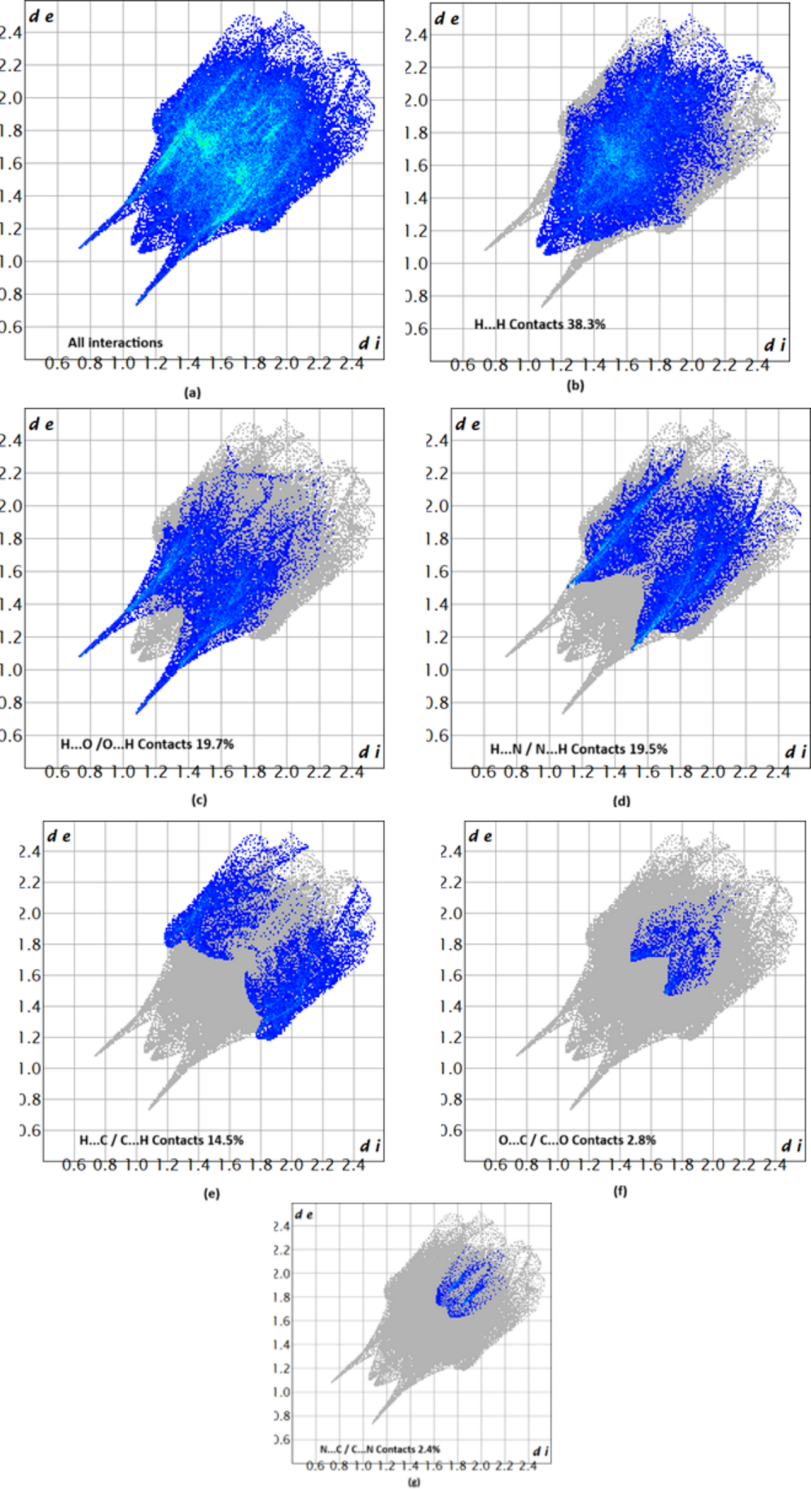
Two-dimensional fingerprint plots for compound (I), showing (*a*) all inter­actions, and delineated into (*b*) H⋯H, (*c*) H⋯O/O⋯H, (*d*) H⋯N/N⋯H, (*e*)H⋯C/C⋯H, (*f*) O⋯C/C⋯O and (*g*) N⋯C/C⋯N inter­actions. The *d*_i_ and *d*_e_ values are the closest inter­nal and external distances (in Å) from given points on the Hirshfeld surface.

The associated two-dimensional fingerprint plots (McKinnon *et al.*, 2007 ▸) provide qu­anti­tative information about the non-covalent inter­actions in the crystal packing in terms of the percentage contribution of the inter­atomic contacts (Spackman & McKinnon, 2002 ▸). The overall two-dimensional fingerprint plot is shown in Fig. 5 ▸*a*. The HS analysis reveals that H⋯H and H⋯O/O⋯H contacts are the main contributors to the crystal packing, followed by H⋯N/N⋯H, H⋯C/C⋯H, O⋯C/C⋯O and C⋯N/N⋯C contacts; see Fig. 5 ▸*b*–*g*. The HS analysis confirms the importance of H-atom contacts in the crystal (Hathwar *et al.*, 2015 ▸).

## 5. Synthesis and crystallization

The title compound (I) was synthesized by condensing 2-cyano acetohydrazide in methanol with 3,4,5-tri­meth­oxy­benzaldehyde in a 1:1 ratio following an established protocol (Shaik *et al.*, 2019 ▸). The progress of the reaction was monitored by thin layer chromatography (TLC). After completion of the reaction, methanol was removed under vacuum. The solid product was collected, washed, and recrystallized from methanol to obtain crystals of (I).

## 6. Refinement

Crystal data, data collection and structure refinement details are summarized in Table 2 ▸. All H atoms were placed in idealized positions and allowed to ride on their parent atoms: N—H = 0.86 and C—H = 0.93–0.97 Å, with *U*_iso_(H) = 1.5*U*_eq_(C) for methyl H atoms and *U*_iso_(H) = 1.2*U*_eq_(C, N) for all other H atoms.

## Supplementary Material

Crystal structure: contains datablock(s) I, global. DOI: 10.1107/S2056989025003913/tx2097sup1.cif

Structure factors: contains datablock(s) I. DOI: 10.1107/S2056989025003913/tx2097Isup2.hkl

Supporting information file. DOI: 10.1107/S2056989025003913/tx2097Isup3.cml

CCDC reference: 2448154

Additional supporting information:  crystallographic information; 3D view; checkCIF report

## Figures and Tables

**Table 1:** Hydrogen-bond geometry (Å, °)

*D*—H⋯*A*	*D*—H	H⋯*A*	*D*⋯*A*	*D*—H⋯*A*
C9—H9*B*⋯O2^i^	0.97	2.50	3.448 (3)	165
C13—H13*A*⋯O4^ii^	0.96	2.51	3.350 (3)	146
N2—H2⋯O4^iii^	0.86	1.98	2.802 (3)	160

Symmetry codes: (i) 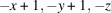; (ii) 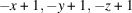; (iii) 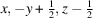.

**Table 2:** Experimental details

Crystal data	
Chemical formula	C_13_H_15_N_3_O_4_
*M*_r_	277.28
Crystal system, space group	Monoclinic, *P*2_1_/*c*
Temperature (K)	298
*a*, *b*, *c* (Å)	13.9944 (8), 11.0371 (7), 9.0560 (5)
β (°)	99.936 (2)
*V* (Å^3^)	1377.79 (14)
*Z*	4
Radiation type	Mo *K*α
μ (mm^−1^)	0.10
Crystal size (mm)	0.16 × 0.12 × 0.08

Data collection	
Diffractometer	Bruker APEXII CCD
Absorption correction	Multi-scan (*SADABS*; Krause *et al.*, 2015 ▸)
*T*_min_, *T*_max_	0.624, 0.745
No. of measured, independent and observed [*I* > 2σ(*I*)] reflections	23917, 2823, 1540
*R*_int_	0.072
(sin θ/λ)_max_ (Å^−1^)	0.625

Refinement	
*R*[*F*^2^ > 2σ(*F*^2^)], *wR*(*F*^2^), *S*	0.053, 0.156, 1.05
No. of reflections	2823
No. of parameters	181
H-atom treatment	H-atom parameters constrained
Δρ_max_, Δρ_min_ (e Å^−3^)	0.15, −0.23

Computer programs: *APEX3* and *SAINT* (Bruker, 2017 ▸), *SHELXT2018/2* (Sheldrick, 2015*a* ▸), *SHELXL2019/2* (Sheldrick, 2015*b* ▸), *ORTEP-3 for Windows* (Farrugia, 2012 ▸) and *PLATON* (Spek, 2020 ▸).

## supplementary crystallographic information

### (E)-2-Cyano-N'-(3,4,5-trimethoxybenzylidene)acetohydrazide Crystal data

**Table:**

C_13_H_15_N_3_O_4_	*F*(000) = 584
*M**_r_* = 277.28	*D*_x_ = 1.337 Mg m^−^^3^
Monoclinic, *P*2_1_/*c*	Mo *K*α radiation, λ = 0.71073 Å
*a* = 13.9944 (8) Å	Cell parameters from 4885 reflections
*b* = 11.0371 (7) Å	θ = 2.4–22.9°
*c* = 9.0560 (5) Å	µ = 0.10 mm^−^^1^
β = 99.936 (2)°	*T* = 298 K
*V* = 1377.79 (14) Å^3^	Block, brown
*Z* = 4	0.16 × 0.12 × 0.08 mm

### (E)-2-Cyano-N'-(3,4,5-trimethoxybenzylidene)acetohydrazide Data collection

**Table:**

Bruker APEXII CCD diffractometer	1540 reflections with *I* > 2σ(*I*)
Radiation source: i-mu-s microfocus source	*R*_int_ = 0.072
φ and ω scans	θ_max_ = 26.4°, θ_min_ = 2.4°
Absorption correction: multi-scan (SADABS; Krause *et al.*, 2015)	*h* = −17→15
*T*_min_ = 0.624, *T*_max_ = 0.745	*k* = −13→13
23917 measured reflections	*l* = −11→8
2823 independent reflections	

### (E)-2-Cyano-N'-(3,4,5-trimethoxybenzylidene)acetohydrazide Refinement

**Table:**

Refinement on *F*^2^	0 restraints
Least-squares matrix: full	Hydrogen site location: inferred from neighbouring sites
*R*[*F*^2^ > 2σ(*F*^2^)] = 0.053	H-atom parameters constrained
*wR*(*F*^2^) = 0.156	*w* = 1/[σ^2^(*F*_o_^2^) + (0.0585*P*)^2^ + 0.5215*P*] where *P* = (*F*_o_^2^ + 2*F*_c_^2^)/3
*S* = 1.05	(Δ/σ)_max_ < 0.001
2823 reflections	Δρ_max_ = 0.15 e Å^−^^3^
181 parameters	Δρ_min_ = −0.23 e Å^−^^3^

### (E)-2-Cyano-N'-(3,4,5-trimethoxybenzylidene)acetohydrazide Special details

**Table:**

Geometry. All esds (except the esd in the dihedral angle between two l.s. planes) are estimated using the full covariance matrix. The cell esds are taken into account individually in the estimation of esds in distances, angles and torsion angles; correlations between esds in cell parameters are only used when they are defined by crystal symmetry. An approximate (isotropic) treatment of cell esds is used for estimating esds involving l.s. planes.

### (E)-2-Cyano-N'-(3,4,5-trimethoxybenzylidene)acetohydrazide Fractional atomic coordinates and isotropic or equivalent isotropic displacement parameters (Å^2^)

**Table:**

	*x*	*y*	*z*	*U*_iso_*/*U*_eq_	
O1	0.17230 (13)	0.43960 (19)	−0.1731 (2)	0.0708 (6)	
O2	0.15221 (12)	0.52233 (16)	0.0935 (2)	0.0574 (5)	
O3	0.29283 (12)	0.49755 (17)	0.3328 (2)	0.0586 (5)	
O4	0.73449 (12)	0.30054 (17)	0.34803 (19)	0.0589 (5)	
C7	0.51086 (18)	0.3277 (2)	0.0177 (3)	0.0473 (6)	
H7	0.522223	0.308137	−0.077620	0.057*	
N1	0.57857 (14)	0.31831 (18)	0.1302 (2)	0.0471 (5)	
N3	0.98039 (19)	0.2939 (3)	0.3950 (3)	0.0824 (9)	
C1	0.33918 (18)	0.3770 (2)	−0.0817 (3)	0.0502 (7)	
H1	0.347981	0.349974	−0.175678	0.060*	
C2	0.25037 (18)	0.4247 (2)	−0.0625 (3)	0.0496 (7)	
C3	0.23722 (17)	0.4649 (2)	0.0780 (3)	0.0463 (6)	
C4	0.31260 (18)	0.4556 (2)	0.1992 (3)	0.0458 (6)	
C5	0.40109 (18)	0.4078 (2)	0.1801 (3)	0.0485 (7)	
H5	0.451361	0.401270	0.261518	0.058*	
C6	0.41465 (17)	0.3697 (2)	0.0388 (3)	0.0456 (6)	
N2	0.66924 (14)	0.28820 (18)	0.1029 (2)	0.0446 (5)	
H2	0.678178	0.272351	0.013346	0.054*	
C8	0.74341 (17)	0.2840 (2)	0.2180 (3)	0.0423 (6)	
C9	0.84027 (16)	0.2566 (2)	0.1732 (3)	0.0470 (6)	
H9A	0.841502	0.172809	0.141281	0.056*	
H9B	0.848985	0.307709	0.089430	0.056*	
C10	0.9189 (2)	0.2776 (3)	0.2976 (3)	0.0533 (7)	
C11	0.1728 (2)	0.3876 (3)	−0.3140 (4)	0.0847 (10)	
H11A	0.112939	0.406117	−0.379339	0.127*	
H11B	0.226071	0.419600	−0.355726	0.127*	
H11C	0.179561	0.301281	−0.303628	0.127*	
C12	0.0851 (2)	0.4491 (3)	0.1573 (4)	0.0719 (9)	
H12A	0.028134	0.495629	0.164137	0.108*	
H12B	0.067467	0.379559	0.094810	0.108*	
H12C	0.114932	0.422970	0.255613	0.108*	
C13	0.3689 (2)	0.4940 (3)	0.4573 (3)	0.0677 (8)	
H13A	0.346330	0.525646	0.543898	0.102*	
H13B	0.389969	0.411839	0.476062	0.102*	
H13C	0.422169	0.542265	0.436720	0.102*	

### (E)-2-Cyano-N'-(3,4,5-trimethoxybenzylidene)acetohydrazide Atomic displacement parameters (Å^2^)

**Table:**

	*U*^11^	*U*^22^	*U*^33^	*U*^12^	*U*^13^	*U*^23^
O1	0.0544 (12)	0.0881 (15)	0.0646 (13)	0.0106 (10)	−0.0047 (10)	−0.0052 (11)
O2	0.0438 (10)	0.0538 (11)	0.0771 (13)	0.0103 (9)	0.0176 (9)	0.0081 (9)
O3	0.0502 (11)	0.0717 (13)	0.0553 (12)	0.0109 (9)	0.0128 (9)	−0.0084 (9)
O4	0.0594 (12)	0.0831 (14)	0.0376 (10)	0.0069 (10)	0.0184 (9)	0.0000 (9)
C7	0.0466 (15)	0.0463 (15)	0.0517 (16)	0.0016 (12)	0.0157 (13)	−0.0031 (12)
N1	0.0398 (12)	0.0527 (13)	0.0522 (13)	0.0056 (10)	0.0179 (11)	−0.0005 (10)
N3	0.0639 (17)	0.116 (2)	0.0637 (17)	−0.0227 (16)	0.0017 (14)	0.0100 (16)
C1	0.0499 (16)	0.0494 (15)	0.0522 (16)	0.0017 (12)	0.0110 (14)	−0.0042 (12)
C2	0.0438 (15)	0.0492 (15)	0.0534 (17)	0.0003 (12)	0.0020 (13)	0.0048 (13)
C3	0.0364 (14)	0.0400 (14)	0.0635 (18)	0.0024 (11)	0.0110 (13)	0.0062 (12)
C4	0.0460 (15)	0.0413 (14)	0.0521 (16)	0.0025 (11)	0.0138 (13)	0.0018 (12)
C5	0.0419 (15)	0.0508 (16)	0.0527 (16)	0.0048 (12)	0.0081 (12)	0.0020 (12)
C6	0.0432 (15)	0.0416 (14)	0.0532 (16)	0.0016 (11)	0.0118 (13)	−0.0011 (12)
N2	0.0410 (12)	0.0567 (13)	0.0396 (12)	0.0047 (10)	0.0162 (10)	−0.0017 (10)
C8	0.0432 (15)	0.0455 (14)	0.0407 (15)	0.0005 (11)	0.0141 (12)	0.0024 (11)
C9	0.0421 (15)	0.0551 (16)	0.0447 (15)	0.0009 (12)	0.0105 (12)	0.0021 (12)
C10	0.0477 (17)	0.0650 (18)	0.0497 (17)	−0.0052 (14)	0.0156 (14)	0.0077 (14)
C11	0.076 (2)	0.099 (3)	0.070 (2)	0.0001 (19)	−0.0110 (18)	−0.021 (2)
C12	0.0469 (17)	0.071 (2)	0.101 (2)	−0.0049 (15)	0.0221 (17)	0.0050 (18)
C13	0.072 (2)	0.078 (2)	0.0522 (18)	0.0183 (17)	0.0077 (16)	−0.0050 (15)

### (E)-2-Cyano-N'-(3,4,5-trimethoxybenzylidene)acetohydrazide Geometric parameters (Å, º)

**Table:**

O1—C2	1.359 (3)	C5—C6	1.390 (3)
O1—C11	1.401 (3)	C5—H5	0.9300
O2—C3	1.376 (3)	N2—C8	1.339 (3)
O2—C12	1.434 (3)	N2—H2	0.8600
O3—C4	1.367 (3)	C8—C9	1.511 (3)
O3—C13	1.412 (3)	C9—C10	1.451 (4)
O4—C8	1.219 (3)	C9—H9A	0.9700
C7—N1	1.270 (3)	C9—H9B	0.9700
C7—C6	1.467 (3)	C11—H11A	0.9600
C7—H7	0.9300	C11—H11B	0.9600
N1—N2	1.375 (3)	C11—H11C	0.9600
N3—C10	1.136 (3)	C12—H12A	0.9600
C1—C6	1.384 (3)	C12—H12B	0.9600
C1—C2	1.388 (3)	C12—H12C	0.9600
C1—H1	0.9300	C13—H13A	0.9600
C2—C3	1.389 (4)	C13—H13B	0.9600
C3—C4	1.389 (3)	C13—H13C	0.9600
C4—C5	1.384 (3)		
			
C2—O1—C11	119.5 (2)	O4—C8—N2	123.5 (2)
C3—O2—C12	114.76 (19)	O4—C8—C9	122.4 (2)
C4—O3—C13	117.24 (19)	N2—C8—C9	114.1 (2)
N1—C7—C6	119.7 (2)	C10—C9—C8	110.8 (2)
N1—C7—H7	120.1	C10—C9—H9A	109.5
C6—C7—H7	120.1	C8—C9—H9A	109.5
C7—N1—N2	117.3 (2)	C10—C9—H9B	109.5
C6—C1—C2	120.1 (2)	C8—C9—H9B	109.5
C6—C1—H1	120.0	H9A—C9—H9B	108.1
C2—C1—H1	120.0	N3—C10—C9	179.9 (4)
O1—C2—C1	125.2 (2)	O1—C11—H11A	109.5
O1—C2—C3	114.8 (2)	O1—C11—H11B	109.5
C1—C2—C3	119.9 (2)	H11A—C11—H11B	109.5
O2—C3—C4	120.4 (2)	O1—C11—H11C	109.5
O2—C3—C2	119.6 (2)	H11A—C11—H11C	109.5
C4—C3—C2	119.8 (2)	H11B—C11—H11C	109.5
O3—C4—C5	124.2 (2)	O2—C12—H12A	109.5
O3—C4—C3	115.6 (2)	O2—C12—H12B	109.5
C5—C4—C3	120.2 (2)	H12A—C12—H12B	109.5
C4—C5—C6	119.8 (2)	O2—C12—H12C	109.5
C4—C5—H5	120.1	H12A—C12—H12C	109.5
C6—C5—H5	120.1	H12B—C12—H12C	109.5
C1—C6—C5	120.1 (2)	O3—C13—H13A	109.5
C1—C6—C7	120.5 (2)	O3—C13—H13B	109.5
C5—C6—C7	119.3 (2)	H13A—C13—H13B	109.5
C8—N2—N1	118.96 (19)	O3—C13—H13C	109.5
C8—N2—H2	120.5	H13A—C13—H13C	109.5
N1—N2—H2	120.5	H13B—C13—H13C	109.5
			
C6—C7—N1—N2	−174.8 (2)	O2—C3—C4—C5	−174.0 (2)
C11—O1—C2—C1	10.5 (4)	C2—C3—C4—C5	0.8 (4)
C11—O1—C2—C3	−171.3 (3)	O3—C4—C5—C6	−178.7 (2)
C6—C1—C2—O1	178.0 (2)	C3—C4—C5—C6	0.3 (4)
C6—C1—C2—C3	−0.1 (4)	C2—C1—C6—C5	1.3 (4)
C12—O2—C3—C4	−81.8 (3)	C2—C1—C6—C7	−175.5 (2)
C12—O2—C3—C2	103.3 (3)	C4—C5—C6—C1	−1.4 (4)
O1—C2—C3—O2	−4.3 (3)	C4—C5—C6—C7	175.4 (2)
C1—C2—C3—O2	174.0 (2)	N1—C7—C6—C1	−178.0 (2)
O1—C2—C3—C4	−179.2 (2)	N1—C7—C6—C5	5.1 (4)
C1—C2—C3—C4	−1.0 (4)	C7—N1—N2—C8	176.4 (2)
C13—O3—C4—C5	1.6 (4)	N1—N2—C8—O4	3.2 (4)
C13—O3—C4—C3	−177.5 (2)	N1—N2—C8—C9	−176.8 (2)
O2—C3—C4—O3	5.1 (3)	O4—C8—C9—C10	−11.6 (3)
C2—C3—C4—O3	180.0 (2)	N2—C8—C9—C10	168.4 (2)

### (E)-2-Cyano-N'-(3,4,5-trimethoxybenzylidene)acetohydrazide Hydrogen-bond geometry (Å, º)

**Table:**

*D*—H···*A*	*D*—H	H···*A*	*D*···*A*	*D*—H···*A*
C9—H9*B*···O2^i^	0.97	2.50	3.448 (3)	165
C13—H13*A*···O4^ii^	0.96	2.51	3.350 (3)	146
N2—H2···O4^iii^	0.86	1.98	2.802 (3)	160

Symmetry codes: (i) −*x*+1, −*y*+1, −*z*; (ii) −*x*+1, −*y*+1, −*z*+1; (iii) *x*, −*y*+1/2, *z*−1/2.
